# A Role for Phosphatidic Acid in the Formation of “Supersized” Lipid Droplets

**DOI:** 10.1371/journal.pgen.1002201

**Published:** 2011-07-28

**Authors:** Weihua Fei, Guanghou Shui, Yuxi Zhang, Natalie Krahmer, Charles Ferguson, Tamar S. Kapterian, Ruby C. Lin, Ian W. Dawes, Andrew J. Brown, Peng Li, Xun Huang, Robert G. Parton, Markus R. Wenk, Tobias C. Walther, Hongyuan Yang

**Affiliations:** 1 School of Biotechnology and Biomolecular Sciences, University of New South Wales, Sydney, Australia; 2 Department of Biochemistry, National University of Singapore, Singapore, Singapore; 3 Department of Biological Sciences, National University of Singapore, Singapore, Singapore; 4 Department of Cell Biology, Yale University School of Medicine, New Haven, Connecticut, United States of America; 5 Institute for Molecular Bioscience and Centre for Microscopy and Microanalysis, University of Queensland, Brisbane, Australia; 6 Department of Biological Sciences and Biotechnology, Tsinghua University, Beijing, China; 7 Laboratory of Molecular and Developmental Biology, Institute of Genetics and Developmental Biology, Chinese Academy of Sciences, Beijing, China; University of California San Francisco, United States of America

## Abstract

Lipid droplets (LDs) are important cellular organelles that govern the storage and turnover of lipids. Little is known about how the size of LDs is controlled, although LDs of diverse sizes have been observed in different tissues and under different (patho)physiological conditions. Recent studies have indicated that the size of LDs may influence adipogenesis, the rate of lipolysis and the oxidation of fatty acids. Here, a genome-wide screen identifies ten yeast mutants producing “supersized” LDs that are up to 50 times the volume of those in wild-type cells. The mutated genes include: *FLD1*, which encodes a homologue of mammalian seipin; five genes (*CDS1*, *INO2*, *INO4*, *CHO2*, and *OPI3*) that are known to regulate phospholipid metabolism; two genes (*CKB1* and *CKB2*) encoding subunits of the casein kinase 2; and two genes (*MRPS35* and *RTC2*) of unknown function. Biochemical and genetic analyses reveal that a common feature of these mutants is an increase in the level of cellular phosphatidic acid (PA). Results from *in vivo* and *in vitro* analyses indicate that PA may facilitate the coalescence of contacting LDs, resulting in the formation of “supersized” LDs. In summary, our results provide important insights into how the size of LDs is determined and identify novel gene products that regulate phospholipid metabolism.

## Author Summary

Lipid droplets (LD) are primary lipid storage structures that also function in membrane and lipid trafficking, protein turnover, and the reproduction of deadly viruses. Increased LD accumulation in liver, skeletal muscle, and adipose tissue is a hallmark of the metabolic syndrome. Enlarged LDs are often found in these tissues under disease conditions. However, little is known about how the size of LDs is controlled in eukaryotic cells. In this study, we use genetic and biochemical methods to identify important gene products that regulate the size of the LDs. Notably, a common feature among these mutants with “supersized” LDs is an increased level of phosphatidic acid (PA). We also show that a small amount of PA can increase the size of artificial LDs *in vitro*. Overall, our study identifies important lipids and proteins in determining LD size. These results provide valuable insights into how human cells/tissues handle abnormal influx of lipids in today's obesogenic environment.

## Introduction

Lipid droplets (LDs) are dynamic organelles that govern the storage and turnover of lipids [1]. They also play important roles in membrane and lipid trafficking, protein storage, protein degradation and the replication of hepatitis C and dengue viruses [1], [2], [3], [4], [5]. All LDs comprise a core of storage neutral lipids, i.e. triacylglycerols (TAG) and sterol esters (SE), which are wrapped by a monolayer of phospholipids containing embedded proteins. LDs are believed to originate from the endoplasmic reticulum (ER), although the exact mechanism underlying their biogenesis remains to be determined [6].

LDs of various sizes have been observed in different tissues or within the same cell type under different (patho)physiological conditions [7], [8]. A giant (up to 200 µm in diameter), unilocular LD often occupies the entire cytoplasm of white adipocytes, specializing in energy storage. In contrast, many much smaller LDs (usually less than 10 µm in diameter) are found in brown adipocytes. Small LDs are also found in normal liver cells; however, the size of liver LDs increases dramatically in hepatic steatosis, e.g. in the *ob/ob* mice [9]. The physiological significance of LD size has not been well recognized and little is known about the molecular mechanisms that influence LD size. Recent studies have begun to shed light on the control and physiological relevance of LD size. Deletion of FSP27 (fat-specific protein of 27 kDa) resulted in many smaller LDs in white adipocytes, enhanced lipolysis and protection from diet-induced obesity and insulin resistance [8], [10]. A genome-wide RNA interference (RNAi) screen in *Drosophila* S2 cells identified enzymes of phospholipid biosynthesis as determinants of LD size and number [11]. Interestingly, screens of the viable yeast deletion library found extensive clustering of LDs and formation of “Supersized” LDs (SLDs) that are up to 50 times the normal volume in cells deleted for *FLD1* [12], which encodes a functional homologue of a human lipodystrophic protein: seipin [13], [14].

Congenital generalized lipodystrophy (CGL) is characterized by a nearly complete absence of adipose tissue and a range of metabolic changes such as extreme insulin resistance [15]. Genome-wide linkage analysis identified two loci for CGL: CGL type 1 (CGL1) is caused by mutations in the 1-acylglycerol-3-phosphate-O-acyl transferase 2 (AGPAT2) gene [16] and CGL2 by mutations in BSCL2 which encodes seipin [17]. AGPAT2 catalyzes the formation of phosphatidic acid (PA) but knocking down AGPAT2 led to elevated levels of several phospholipid species including PA [18], [19]. In mice, mutations in the lipin-1 gene which encodes a phosphatidate phosphatase are responsible for severe lipodystrophy [20], [21]. Therefore, both AGPAT2 and lipin-1 appear to control adipogenesis through modulation of the synthesis of phospholipids and triacylglycerol precursors, especially PA. In contrast, although Fld1p (yeast seipin) has been implicated in lipid metabolism, little information is available on its molecular function [12], [22].

Previous genome-wide studies of yeast LDs covered only non-essential yeast genes and used only one culture condition [12], [13]. Here, a revised screen of yeast deletion mutants for the formation of “supersized” lipid droplets (SLDs) identifies known and novel proteins in phospholipid metabolism. These mutants including *fld1Δ* share one common feature: an increase in the level of PA. Reducing the amount of PA invariably led to a significant reduction in SLD formation in all mutants. Finally, a critical role of PA in LD coalescence is confirmed when LD formation is reconstituted *in vitro*.

## Results

### Identification of additional yeast mutants with “supersized” LDs

**Figure 1:**
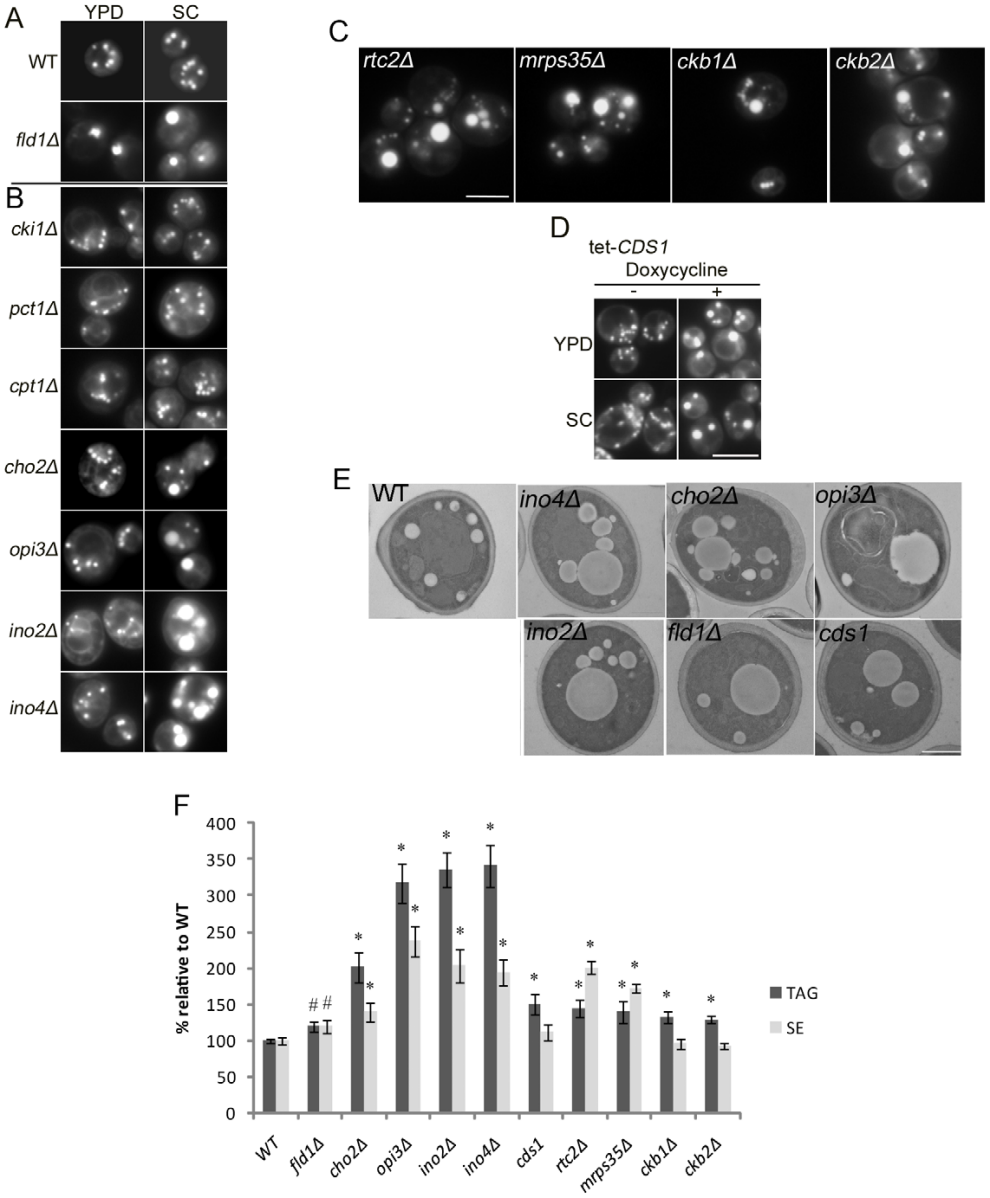
Yeast *fld1Δ* and nine additional mutant strains produce “supersized” LDs (SLDs). Cells were grown in YPD or SC medium until early stationary phase, stained with Nile red, and examined by fluorescence microscopy. Bar, 5 µm. A). LDs of WT and *fld1Δ*. B). LDs of mutant strains defective in either CDP-choline pathway (*cki1Δ*, *pct1Δ*, and *cpt1Δ*) or PEMT pathway (*cho2Δ*, *opi3Δ*, *ino2Δ*, and *ino4Δ*) of PC synthesis. C). Supersized LDs observed in *rtc2Δ*, *mrps35Δ*, *ckb1Δ* and *ckb2Δ*. D). LDs of a yeast strain with the *CDS1* gene under the control of a tetracycline-regulated promoter grown in the presence or absence of doxycycline. E). Transmission electron microscopic examination of LDs in WT, *fld1Δ*, *cho2Δ*, *opi3Δ*, *ino2Δ*, *ino4Δ*, and *cds1* strains cultured in SC medium. Bar, 1 µm. F). Relative cellular amounts of TAG and SE. #, *p*<0.05; *, *p*<0.01, compared to WT.

We previously identified yeast gene deletions that led to a reduced number of cytoplasmic LDs, and we noticed that one of these mutants, *fld1Δ*, developed very large LDs (Figure 1A and [12]). Whereas the diameter of LDs in wild type (WT) cells typically ranges between 0.3 to 0.4 µm, and rarely exceeds 0.5 µm [23], *fld1Δ* cells often synthesize LDs with a diameter larger than 1.0 µm [12]. We arbitrarily define LDs with a diameter greater than 1.0 µm as “supersized” LDs (SLDs), whose volume is over 30 times the average of wild type LDs. About 20% of *fld1Δ* cells cultured in rich (YPD) medium contained SLDs, and the percentage increased to ∼70% when cells were grown in minimal (synthetic complete/SC) medium (Figure 1A and Table 1).

**Table 1:** Yeast gene deletions that lead to the formation of supersized LDs (SLDs).

SLD	ORF	Gene	Function	% of cells with SLDs*	
				SC media	YPD media
1	YLR404W	*FLD1, SEI1*	Unknown	66.1±1.0	22.3±3.7
2	YGR157W	*CHO2*	PE methyltransferase	60.9±5.8	0.5±0.5
3	YJR073C	*OPI3*	Phospholipid methyltransferase	89.3±3.2	0.7±0.3
4	YDR123C	*INO2*	Transcription factor	97.0±1.0	0.3±0.3
5	YOL108C	*INO4*	Transcription factor	96.3±1.6	0.5±0.0
6	YBR147W	*RTC2*	Unknown	71.0±2.7	0.7±0.3
7	YGR165W	*MRPS35*	Unknown	69.3±5.1	0.3±0.3
8	YGL019W	*CKB1*	Beta regulatory subunit of casein kinase 2	32.8±3.4	0.5±0.5
9	YOR039W	*CKB2*	Beta' regulatory subunit of casein kinase 2	20.0±3.2	0.7±0.3
10	YBR029C	*CDS1*	CDP-DAG synthase	46.6±3.7	29.1±5.6
WT				0.7±0.3	0.3±0.3

Cells were grown in synthetic complete (SC) media or rich YPD media to stationary phase, stained with Nile red, and observed under a fluorescence microscope for LDs. Experiments were done in triplicates and ∼200 cells were counted for all strains each time. The percentages of cells that displayed SLDs were represented as mean±SD. SC has 11 µM inositol because it contains yeast nitrogen base.

Given the effects various nutrients may have on the dynamics of LDs, we reasoned that growing cells on defined, minimal media (SC) would reduce the impact of nutrients and uncover additional genes. We have therefore screened the entire collection of viable yeast deletion mutants (∼4800) grown on minimal (SC) media for SLDs. In addition, our previous screen focused only on the viable yeast deletion mutants, representing ∼80% of the genome. In order to identify essential genes impacting LD size, we now included the collection of mutants where all essential genes are controlled by the TetO_7_-promoter, which can be switched off efficiently [24]. Besides *fld1Δ*, we identified nine additional mutants (*sld2-10*) that produced supersized LDs (SLDs) (Figure 1 and Table 1). Except for two previously uncharacterized genes (*RTC2*/*SLD6&MRPS35/SLD7*), the majority of the *SLD* genes appear to function directly or indirectly in the metabolism of phospholipids, especially phosphatidylcholine (PC). PC is synthesized in yeast via two pathways: the Kennedy pathway and the phosphatidylethanolamine N-methyltransferase (PEMT) pathway (see Figure S1). In the PEMT pathway, phosphatidylethanolamine (PE) is methylated to PC in three steps by two methyltransferases, Cho2p and Opi3p. Besides *cho2Δ* and *opi3Δ* mutants, *ino2Δ* and *ino4Δ* mutants are also defective in PC synthesis via PE methylation since Ino2p and Ino4p are transcription factors that positively regulate the PEMT pathway [25]. When *cki1Δ*, *pct1Δ*, *cpt1Δ*, *cho2Δ*, *opi3Δ*, *ino2Δ*, and *ino4Δ* cells were cultured in rich (YPD) medium, none of these mutants accumulated SLDs. In contrast, when grown in SC medium (no choline and hence little Kennedy pathway activity), approximately 60% of *cho2Δ* cells, 90% of *opi3Δ* cells, 97% of *ino2Δ* and *ino4Δ* cells produced SLDs, whereas *cki1Δ*, *pct1Δ*, and *cpt1Δ* cells did not (Figure 1B and 1E). From these results, PC synthesis does appear to be critical in regulating the size of LDs, in agreement with a recent study examining LD dynamics in *Drosophila* S2 cells [11]. Interestingly, of the 825 essential genes examined, SLDs were observed only upon knocking-down *CDS1* (encoding CDP-diacylglycerol synthase) (Figure. 1D and 1E). Therefore, the synthesis of not only PC, but also other phospholipids could be important for LD growth. The SLDs observed in *fld1Δ, cho2Δ, opi3Δ, ino2Δ, ino4Δ,* and TetO_7_-*CDS1* (thereafter referred to as *cds1* when repressed by doxycycline) strains were further confirmed by electron microscopy (Figure 1E). The levels of TAG and SE of all mutants were also examined, and the level of TAG was significantly increased in all mutants (Figure 1F).

### Defective TAG mobilization of “supersized” LDs

As compared to many small LDs in WT cells, the formation of SLDs limits the surface area that is accessible to lipases. Therefore, the mobilization of TAG may be impaired in *sld* mutants. TAG breakdown in *fld1Δ*, *ino4Δ*, and *cds1* strains was monitored in the presence of 10 mg/L cerulenin that prevents their *de novo* synthesis as described [26]. Our results show that TAG mobilization in the mutants is significantly slower than that of WT (Figure S2).

### Treatment of different phospholipid precursors exerted distinct effects on the formation of SLDs

**Figure 2:**
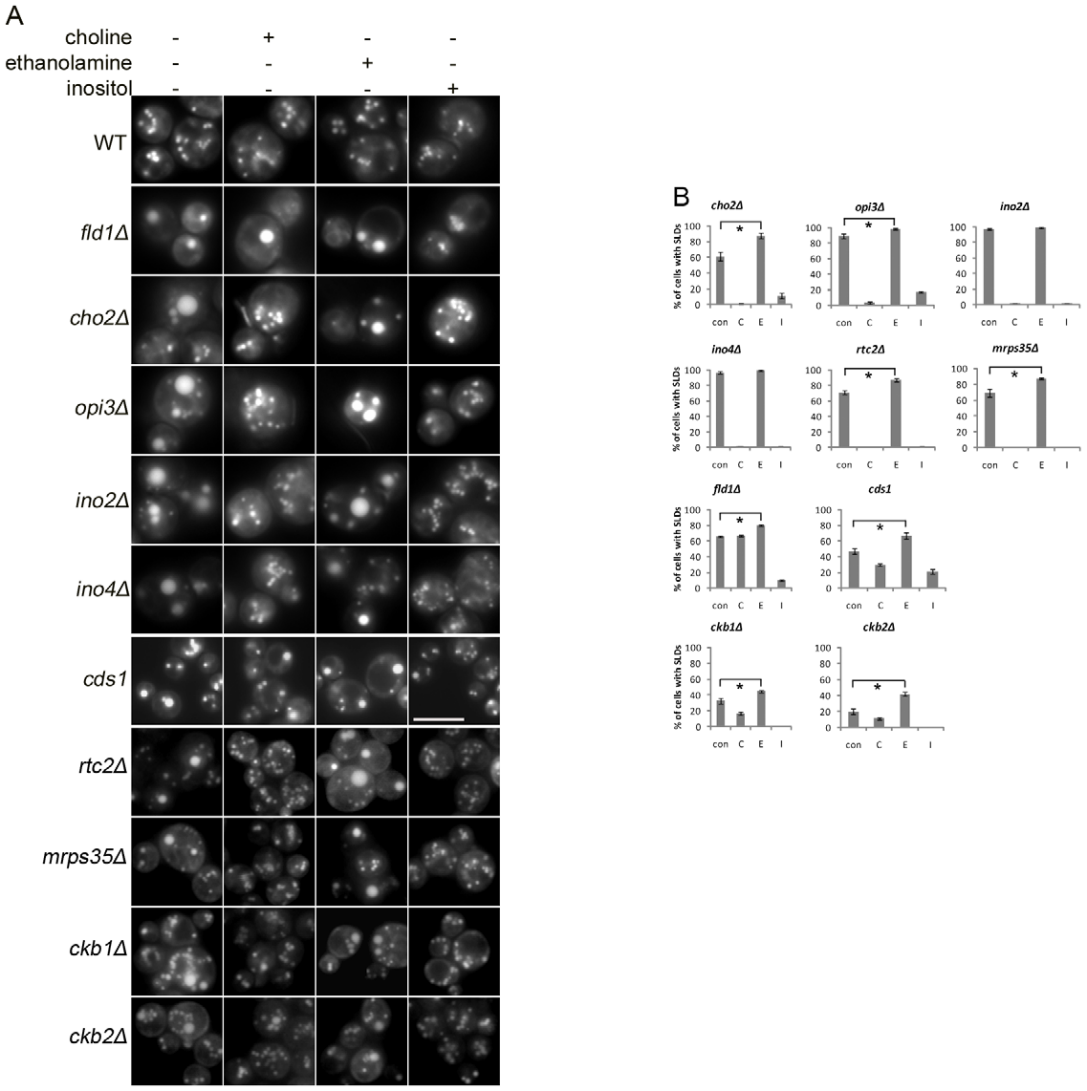
Treatment of different phospholipid precursors exerts distinct effects on the formation of SLDs. WT and mutants were cultured in SC media supplemented without (con) or with 1 mM choline (C), 1 mM ethanolamine (E), or 75 µM inositol (I) to stationary phase and observed under a fluorescence microscope. A). Microscopic images. Bar, 5 µm. B). Percentage of cells containing SLDs. *, *p*<0.01.

Our finding that YPD media invariably decreased the percentage of cells displaying SLDs in all *sld* mutants suggested that certain components present in rich YPD media, but absent or low in SC media, suppressed SLD formation. Considering that Cds1p, Cho2p, Opi3p, Ino2p, and Ino4p are either enzymes or transcription factors involved in phospholipid biosynthesis, we speculated that these components might be precursors of phospholipids. To examine this possibility, we cultured WT and mutant strains in SC media supplemented with 1 mM choline, 1 mM ethanolamine, or 75 µM inositol. Interestingly, inositol treatment reduced the SLD formation in all mutants (Figure 2). In contrast, ethanolamine addition had an opposite effect; it enhanced SLD formation in most of the mutants. As expected, choline addition completely blocked the formation of supersized LDs in *cho2Δ*, *opi3Δ*, *ino2Δ* and *ino4Δ* strains (Figure 2A and 2B), since exogenously added choline restored PC synthesis in these mutants through the Kennedy pathway. Surprisingly it also had similar effect in *rtc2Δ* and *mrps35Δ* strains, suggesting that these two genes may also function in PC metabolism. Choline addition also partially inhibited SLD formation in *cds1*, *ckb1Δ*, and *ckb2Δ* cells, but had little effect in *fld1Δ* cells.

**Figure 3:**
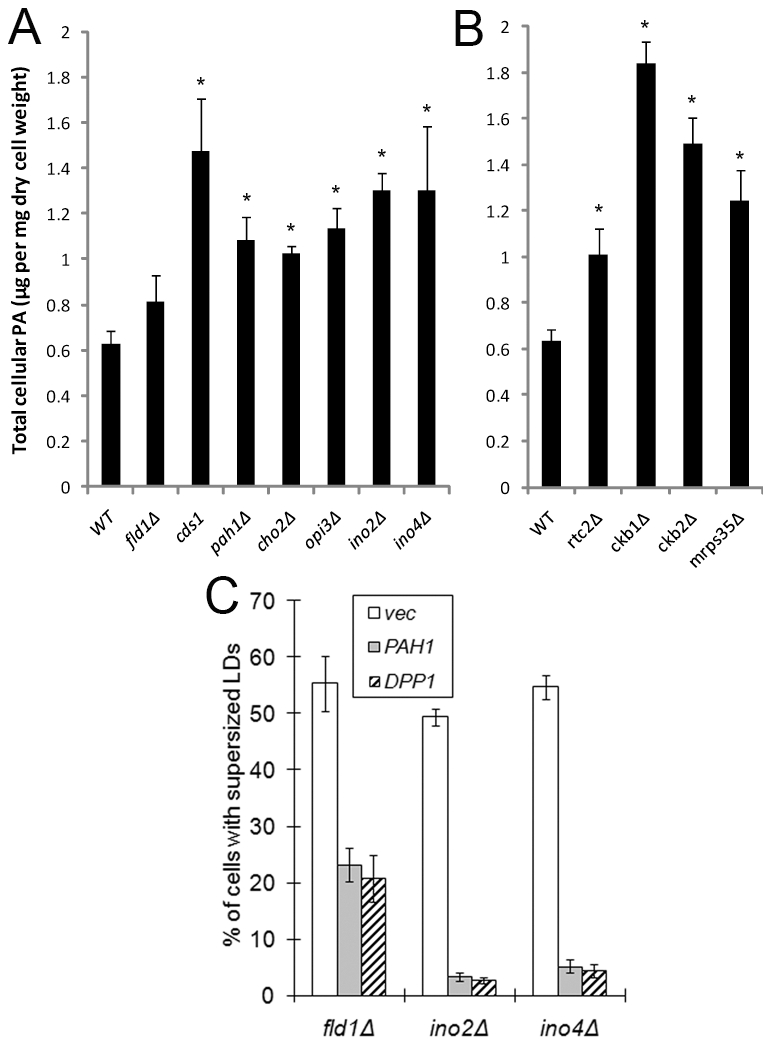
A link between the formation of SLDs and an elevated level of cellular PA. A&B). Quantitation of cellular PA in WT and mutants. Cells were grown in SC medium to early stationary phase, harvested, and lyophilized. Lipids were extracted and PA levels were determined by LC-MS. *, *p*<0.05, compared to WT. C). Overexpression of *PAH1* and *DPP1* significantly reduces the formation of SLDs in *fld1Δ*, *ino2Δ*, and *ino4Δ* strains. Cells transformed with *BG1805-PAH1*, *BG1805-DPP1* (both from Open Biosystems) or empty vector were cultured in synthetic galactose medium (2% galactose, 0.67% yeast nitrogen base, and amino acids) to stationary phase, stained with Nile red, and examined for the presence of SLDs.

### A link between the generation of “supersized” LDs and an elevated level of intracellular phosphatidic acid (PA)

One notable common feature among *cho2Δ, opi3Δ, ino2Δ, ino4Δ* and *cds1* mutants is the accumulation of PA (Figure 3A and [25], [27]). PA is a cone-shaped lipid that alters the curvature of the membranes, and has been shown to promote both SNARE-dependent and -independent membrane fusion events [28], [29]. A previous study implicated PA in the assembly of lipid droplets from newly synthesized TAGs in a cell-free system [30]. To examine whether PA is a key player in the formation of SLDs, we first analyzed the cellular level of PA in the SLD mutants by LC-MS. Indeed a significant elevation of PA was seen in all mutants except *fld1Δ* cells, where the level of PA is only moderately elevated (Figure 3A and 3B). Inositol treatment reduces the cellular PA pool through increased synthesis of phosphatidylinositol (PI) and also through the activation of a Mg^2+^-dependent PA phosphatase [25], [31]. Consistent with the implication of PA in SLD formation, inositol treatment resulted in a significant reduction of SLD formation in all mutants including *fld1Δ* (Figure 2). In addition, when two PA phosphatases (*PAH1* and *DPP1*) were overexpressed under a *GAL1* promoter [20], [32], both greatly reduced SLD formation in *ino2Δ* and *ino4Δ* cells, and also in *fld1Δ* cells (Figure 3C). Overexpression of *PAH1* and *DPP1* did not change the level of PC, PE, PS and PI, but significantly reduced the cellular level of PA in *fld1Δ* cells (Figure S3). These results imply that the increased amount of PA may account for the formation of SLDs, and that the level of PA in subcellular organelles such as the endoplasmic reticulum where LDs originate may have changed in *fld1Δ* cells, despite an insignificant increase in overall PA in this mutant (see below).

### Fld1p could regulate the metabolism of phospholipids

**Figure 4:**
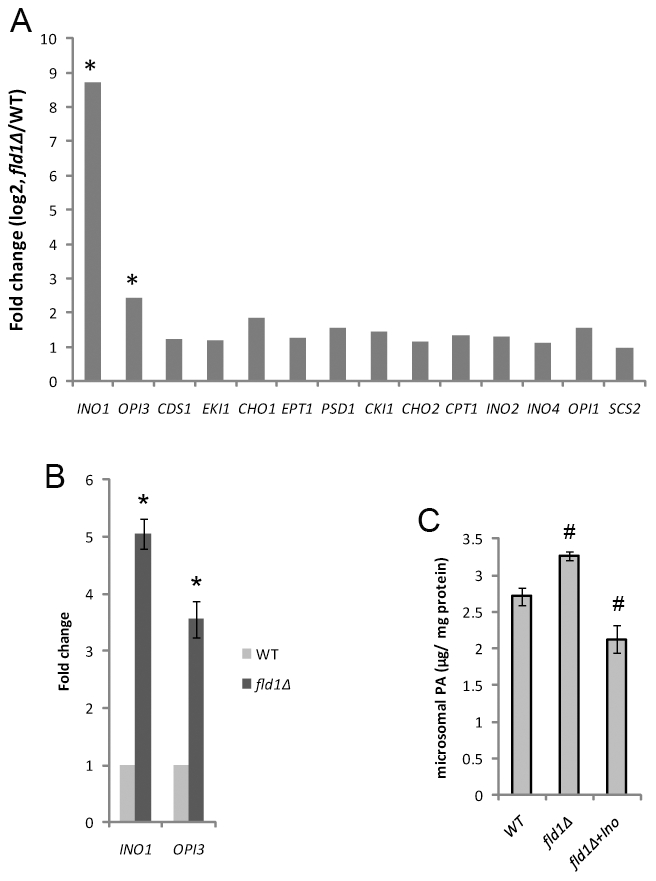
Fld1p and cellular PA. A) The gene expression fold changes (*fld1Δ*/WT) for selective genes involved in phospholipid metabolism as determined by microarray analysis. Cells were cultured in YPD medium until log phase (OD_600_∼0.8). The expression levels of *INO1* and *OPI3* were significantly upregulated in *fld1Δ* cells. *, *p*<0.01 (ANOVA, FDR <0.05). B) Relative mRNA levels of *INO1* and *OPI3* as determined by qPCR in WT and *fld1Δ* strains and normalized to *ACT1*. *, *p*<0.01. C) WT and *fld1Δ* cells without or with (+Ino) inositol treatment were grown to late log phase. Microsomes were isolated as described in methods. Lipids were extracted and the amounts of PA were determined by mass-spectrometry. #, *p*<0.05, compared to WT.

SLDs in yeast were originally identified in *fld1Δ* cells; but the molecular function of Fld1p/seipin remains elusive [22]. To gain more insights into the function of Fld1p, mRNA microarray analysis was performed in WT and *fld1Δ* cells. Of ∼5800 transcripts examined, *INO1* and *OPI3* were the only transcripts whose levels were significantly upregulated in *fld1Δ* cells (Figure 4A). Quantitative real-time PCR confirmed a ∼5-fold increase in the *INO1* mRNA level in *fld1Δ* cells (Figure 4B). *INO1* gene expression is derepressed when intracellular PA concentration rises [25]. We therefore examined the level of PA on the ER where the Opi1p-Scs2p regulatory complex of *INO1* expression exists [33]. Indeed, a significant increase of PA was observed in microsomes isolated from *fld1Δ* cells (Figure 4C). These results show that Fld1p, as other *SLD* mutants, can regulate PA metabolism, and also suggest that both the level and location of PA are relevant to droplet formation. A recent study in *Drosophila* also revealed a possible role for dSeipin in PA metabolism [34].

### Elevated PA enhances SLD formation in yeast

**Figure 5:**
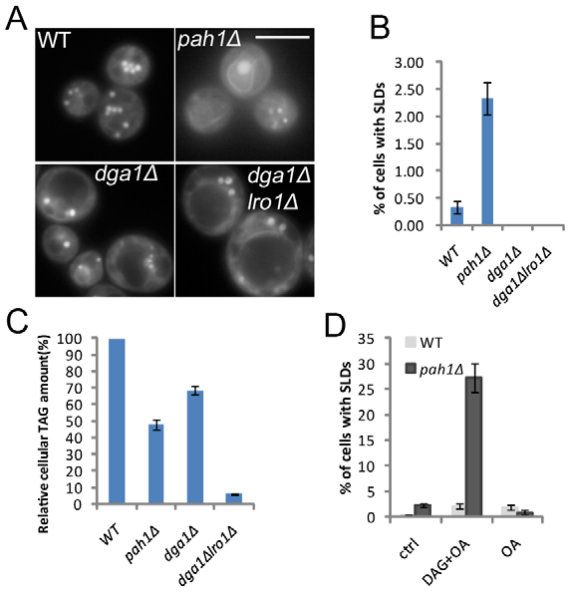
SLD formation in yeast cells deficient in PA phosphatase activity. A&B) Formation of SLDs in *pah1Δ*, but not in *dga1Δ* or *dga1Δ lro1Δ* strains. Bar, 5 µm. C) Relative cellular TAG amounts quantified by thin layer chromatography and densitometry. D) Addition of 1 mM membrane-permeable DAG analog (1,2-dioctanoyl-sn-glycerol) and 1 mM oleate, but not oleate alone, significantly elevated the percentage of *pah1Δ* cells accumulating supersized LDs. Cells with supersized LDs were counted and the percentages were presented as mean±SD.

Another strategy to increase PA is through inactivation of Pah1p, the PA phosphatase and ortholog of mammalian lipin proteins [20], [21]. Deletion of *PAH1* leads to a dramatic increase in the level of its substrate, PA, but causes a dramatic reduction in the amount of TAG [20]. Although the number of LDs was significantly reduced in *pah1Δ* cells, LD size was comparable to that in WT cells. Remarkably, SLDs were detected consistently in ∼3% of *pah1Δ* cells, though its TAG synthesis was decreased by over 50% (Figure 5A–5C). In contrast, no SLDs were ever observed in *dga1Δ lro1Δ* cells, which have little diacylglycerol (DAG) acyltransferase activity [35], [36]. Interestingly, when *pah1Δ* cells were supplemented with oleate and DAG which bypasses the lack of PA phosphatase activity, the number of cells producing SLD increased to ∼30%, whereas oleate alone had no effect (Figure 5D). These results further indicate that PA plays an important role in SLD formation, and this role is more pronounced when the biosynthesis of TAG is not severely compromised.

### Elevated phosphatidylethanolamine concentration is a factor in the biogenesis of SLDs

**Figure 6:**
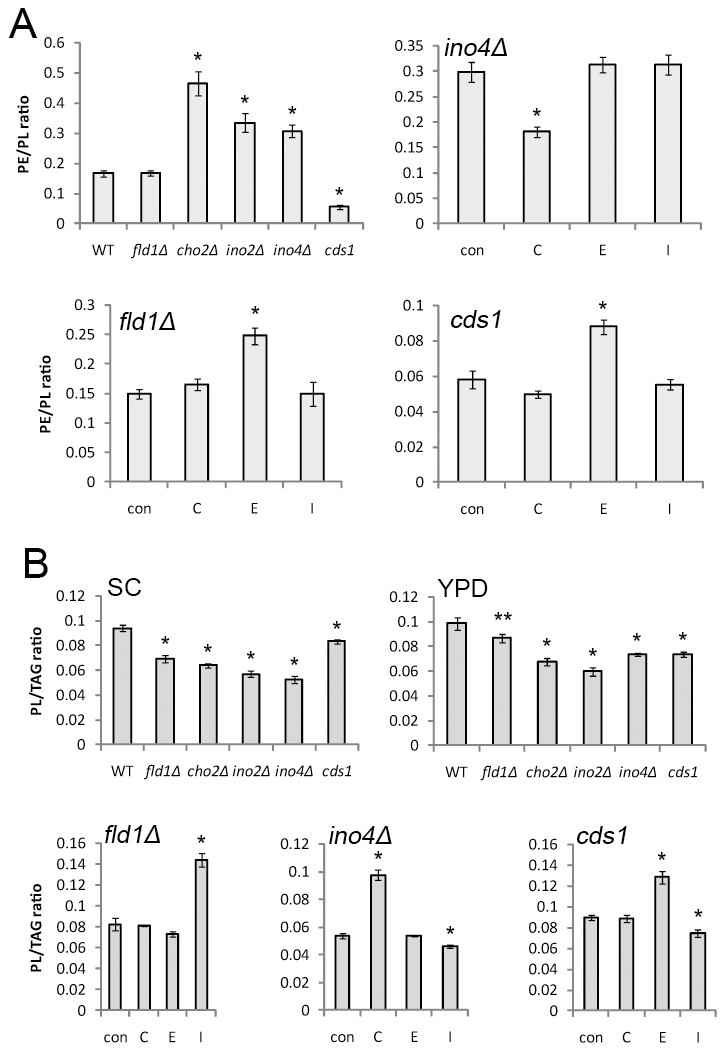
PE/PL and PL/TAG ratios of LDs. A) PE/PL ratio of LDs isolated from WT and mutants grown in SC medium, and of LDs isolated from *ino4Δ*, *fld1Δ*, and *cds1* cells cultured in SC medium without (con) or with the addition of choline (C), ethanolamine (E), or inositol (I). B) PL/TAG ratio of LDs isolated from WT and mutants grown in SC medium or YPD medium, and of LDs isolated from *ino4Δ*, *fld1Δ*, and *cds1* cells cultured in SC medium without (con) or with the addition of choline (C), ethanolamine (E), or inositol (I). *, *p*<0.01; **, *p*<0.05, compared to WT.

The result that ethanolamine addition enhanced SLD formation in nearly all mutants (Figure 2) suggested that an elevated phosphatidylethanolamine (PE) concentration could have a role in SLD formation, given that PE is also a cone-shaped phospholipid that can increase membrane curvature, thereby promoting LD monolayer coalescence [11]. Consistent with this notion, mutants known to accumulate PE also displayed a higher percentage of cells forming SLDs, particularly *opi3Δ*, *ino2Δ*, and *ino4Δ* (Table 1). As shown in Figure 6A, our lipidomic analysis further revealed that lipid droplets isolated from *cho2Δ*, *ino2Δ*, and *ino4Δ* also had a higher PE to PL (total membrane phospholipids) ratio than those of WT cells. In addition, ethanolamine treatment significantly increased the proportion of PE on lipid droplets of *fld1Δ* and *cds1* cells. Even in LDs of *ino4Δ* cells, ethanolamine addition still moderately increased the PE to PL ratio, though its PE level was already much higher than WT. However, elevated PE alone was not able to induce SLD formation since inositol addition completely abolished the biogenesis of supersized LDs despite that a higher PE to PL ratio persisted in *ino4Δ* cells (Figure 6A). Moreover, SLDs were abundant in *cds1* where the PE/PL ratio was lower than that of the WT (Figure 6A and Figure S4).

### Reduced PL-to-TAG ratio does not completely correlate with the formation of SLDs

A decreased phospholipid (PL) to TAG ratio could also induce SLD formation, since coalescence may be induced to decrease the surface-to-volume ratio of droplets when phospholipids are limiting [11]. This model appears to be true for the mutants grown in SC media. However, when grown in YPD media, SLDs disappeared in *cho2Δ*, *ino2Δ* and *ino4Δ* strains but the decreased PL to TAG ratio persisted (Figure 6B). In addition, inositol supplementation did not increase the phospholipid to TAG ratio in *cds1* or *ino4Δ* mutant (Figure 6B).

### Rtc2p and Mrps35p, new players in phospholipid metabolism

**Figure 7:**
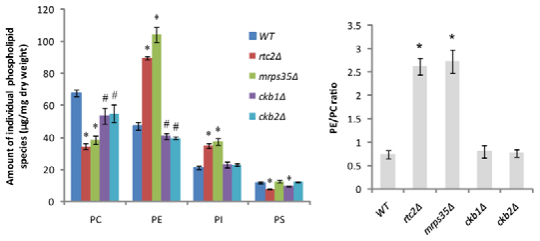
*rtc2Δ* and *mrps35Δ* strains exhibit a higher PE/PC ratio. WT and mutants were cultured in SC medium to early stationary phase. Cellular amounts of major phospholipid species were analyzed by LC-MS. #, *p*<0.05; *, *p*<0.01, compared to WT.

Choline addition completely inhibited SLD formation in *rtc2Δ* and *mrps35Δ*, in a manner similar to *cho2Δ*, *opi3Δ*, *ino2Δ*, and *ino4Δ*, strains known to be defective in the methylation of PE into PC (Figure 2). This phenotype suggests that deletion of *RTC2* or *MRPS35* might affect PC synthesis through the PEMT pathway. As expected, lipidomic analysis revealed that *rtc2Δ* and *mrps35Δ* strains displayed a 2.5-fold increase of PE to PC ratio, indicating these two gene products are involved in PC synthesis through the PEMT pathway (Figure 7). *rtc2Δ* and *mrps35Δ* cells also synthesized ∼60% more phosphatidylinositol (PI) than WT, possibly resulting from the accumulation of CDP-DAG due to a blocked PEMT pathway (Figure 7 and Figure S4A). We also examined the phospholipid profiles of *ckb1Δ* and *ckb2Δ* strains and found that both synthesized less PC and PE than WT without causing significant changes in the PE to PC ratio (Figure 7 and Figure S4A).

### LDs of *sld* mutants demonstrated enhanced fusion activities both *in vivo* and *in vitro*

**Figure 8:**
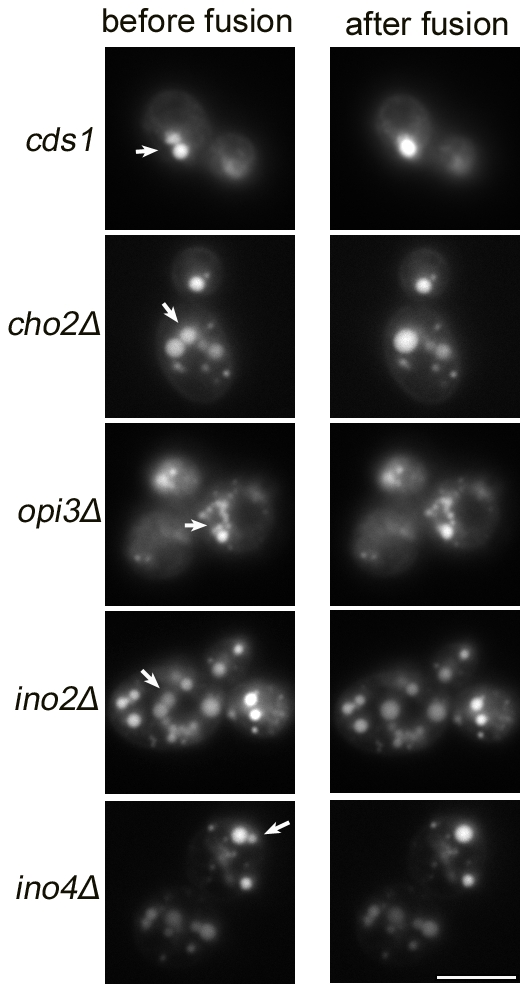
The mutant strains that produce SLDs display enhanced LD fusion activity *in vivo*. Cells were grown in SC media until mid-log phase (OD_600_∼1.5), stained with Nile red and examined for LD fusion activities under fluorescence microscope. Cells in which two or several LDs lay close together were targeted. Images were taken at a 0.5 s interval. Bar, 5 µm.

We next investigated how changes in phospholipids in the *sld* mutants may lead to the formation of SLDs. One possibility could be enhanced fusion activities as previously suggested [12]. Indeed, fusion of Nile red-stained LDs could be observed in *cho2Δ*, *opi3Δ*, *ino2Δ*, *ino4Δ*, and *cds1* strains (Figure 8, Videos S1 and S2), and also in *rtc2Δ, mrps35Δ, ckb1Δ and ckb2Δ* strains (not shown). The fusion frequency of LDs in each mutant was similar (∼10 out of 200 adjacent pairs of LDs) to that of *fld1*Δ [12]. Furthermore, LDs isolated from representative strains demonstrated fusion activities *in vitro* (Videos S3 and S4). It should be noted that no fusion events in wild type yeast cells were ever observed with the methods employed.

### Phosphatidic acid induces fusion of artificial LDs in vitro

**Figure 9:**
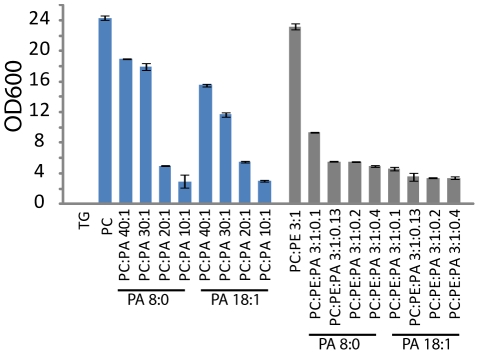
Stability and size of TAG containing artificial droplets consisting of the indicated molar ratios of the phospholipids were determined by light scattering. The artificial droplets were generated by sonication, and liposomes formed at the same time were removed by density gradient centrifugation. The stability/number of LDs was measured by light scattering. Values are mean ± SD of three experiments.

To obtain direct evidence that PA induces coalescence of small LDs to form supersized droplets, artificial LDs were made and their stability tested. After generating the artificial droplets by sonication, we removed liposomes formed at the same time by density gradient centrifugation. This fractionation also concentrated the artificial droplets. From this starting point, we followed the stability of LDs by light scattering, which directly measures the number of LDs. When we increased the concentration of PA in artificial LDs, their number decreased significantly during incubation (Figure 9). In the presence of PE, a smaller fraction of PA (∼3% molar ratio, PC:PE:PA 3∶1∶0.13) achieved a similar effect compared to the coalescence observed in the presence of PC covered LDs (∼5% PA (PC:PA 20∶1). These results show that PA reduces the stability of LDs and mediates coalescence of LDs, and that this property of PA may be modulated by the phospholipid composition of LDs.

## Discussion

Lipid droplets are dynamic organelles whose number and size undergo constant changes in response to internal and external cues [1], [3]. The physiological relevance of the size of the LDs is not well understood, and far less is known about how the size of LDs is determined at the molecular level. In this study, we identify key proteins that govern the size of LDs in yeast by modulating phospholipid metabolism. We also identify proteins previously unknown to regulate phospholipid metabolism. Most importantly, we provide *in vivo* and *in vitro* evidence that phosphatidic acid can influence the size of the LDs.

SLDs provide an efficient form of fat storage in terms of surface to volume ratio. Do cells automatically generate SLDs upon lipid loading to economize on the synthesis of phospholipids that form the surface of lipid droplets? While large lipid droplets are typical in white adipocytes, most other cell types (brown adipocytes, hepatocytes, myocytes) store lipids in numerous small LDs. In WT yeast cells, a dramatic increase in the number but not size of LDs is often observed in growth conditions favoring neutral lipid synthesis and storage, such as starvation. Maintaining small LDs may be physiologically important: upon starvation, yeast cells convert phospholipid intermediates and sterols to neutral lipids which in turn can be hydrolyzed to release fatty acids and sterols for immediate membrane synthesis and cell growth when glucose becomes available. It has also been recently reported that lipolysis is important for efficient cell-cycle progression in yeast [26], and lipolysis occurs more efficiently for small LDs (Figure S2 and [8], [11]). Therefore, it appears that SLDs are only formed in highly specialized, non-dividing cells (e.g. fully differentiated white adipocytes) or under pathological conditions such as severe hepatic steatosis.

Genetic factors that regulate the size of LDs were identified in two separate screens in yeast and in *Drosophila* S2 cells [11], [12]. Decreased PC synthesis, and consequently an increased PE to PL ratio (or a decrease in PC/TAG), has been associated with SLD formation (Figure 1) [11]. LDs are phase-separated organelles in the cytoplasm. Thus, unlike for other organelles, the steady state and lowest energy state is probably to have only one droplet (this would minimize interfacial surface energy). Some phospholipids (specifically PC) shield droplets from coalescence. Fusogenic lipids such as PA and PE could overcome this effect. In agreement with this notion, we found that PE and PA both have an effect on SLD formation. First, treatment of ethanolamine further increased PE to PL ratio and enhanced SLD formation in most mutants (Figure 2 and Figure 6); in addition, strains with increased levels of both PE and PA also had a higher percentage of cells producing supersized LDs (Table 1). Decreased levels of PLs also could lead to SLD formation, since phospholipids levels may not be sufficient under these conditions to prevent the hydrophobic TAG phases to fuse. If the amount of PLs on LDs is not sufficient, fusion would occur until the surface-to-volume ratio of LDs is reflecting the ratio of phospholipids to TAG. At this point the monolayer would shield the LD from any further fusion.

Identification of the *cds1* mutant through the screening of the knock-down collection of essential yeast genes turned our attention to PA, whose critical role in SLD formation was confirmed by the strong “size-reduction” effect of inositol supplementation, an efficient and reliable way to reduce PA in yeast. The essential role of PA in SLD formation was further confirmed when all mutants that develop SLDs were found to accumulate PA (Figure 3), including the seipin-deficient (*fld1Δ*) (Figure 4) and lipin-deficient (*pah1Δ*) (Figure 5A and 5B) cells. PA is a central intermediate in the synthesis of major glycerolphospholipids and TAG, as well as an important signaling lipid [25], [37], [38]. Different pools of PA and distinct PA subclasses may account for the diversity of PA function. For instance, the yeast Opi1p (ER localized transcription repressor) senses only a PA pool on the ER but not the plasma membrane PA pool regulated by the yeast phospholipase D Spo14p [33]. Deleting or overexpressing *SPO14* did not have any impact on the formation of SLDs (data not shown), suggesting that an intracellular (ER/LD) PA pool is responsible for SLD formation. This appears to be also the case for the *fld1Δ* mutant, in which the level of microsomal PA, but not overall PA, is significantly increased. The level of PA on the ER could very well reflect the amount of PA on the LDs, given that LDs are believed to originate from the ER. In summary, we find here that increased PA levels may overcome the effect of phospholipid shielding, and that the location of PA also matters.

Besides establishing a strong link between PA and the size of LDs, our screen also reveals that Rtc2p and Mrps35P can regulate the PEMT pathway of PC synthesis. Both Rtc2p and Mrps35p were found to associate with mitochondria by proteomic studies [39], [40]. We are currently undertaking experiments to understand the role of these two proteins in phospholipid metabolism. Exactly how *ckb1Δ* and *ckb2Δ* mutants cause a significant increase in cellular PA and thereby the formation of SLDs is not clear, although the key transcription factor that regulates phospholipid synthesis in the yeast, Opi1p, can be phosphorylated and regulated by casein kinase 2 [41].

Finally, the mechanistic link between changes in the level of PA/PE and the formation of SLDs was investigated. LDs are covered by a monolayer of phospholipids, whose composition may have a profound effect on the dynamics of the LDs. Both PA and PE are cone-shaped, fusogenic lipids that can alter the curvature of the membranes. PA, in particular, has been shown to promote both SNARE dependent and independent membrane fusion events [28], [29]. It is possible that a higher level of PA on the monolayer of the LDs would promote spontaneous fusion of contacting LDs. Indeed, in vivo microscopic observation found increased incidents of LD fusion in mutants with increased level of PA (Figure 8 and Videos S1, S2, S3, S4). Remarkably, when artificial LDs are made with different ratios of PA, PE and PC, it is clear that even a small amount of PA could significantly increase the size of the LDs (Figure 9).

In summary, studies described herein identify novel protein and lipid regulators of the size of the LDs, an important lipid-storage organelle. Knowing how LD size is determined may provide invaluable insights into how human cells/tissues handle abnormal influx of lipids in today's obesogenic environment.

## Materials and Methods

### Yeast strains

*S. cerevisiae* wild type strain BY4741 (MATa; *his3Δ1; leu2Δ0; met15Δ0; ura3Δ0*) and its derived non-essential gene-deletion strains were either obtained from EUROSCARF or generated in this study. The latter included *pah1Δ* (*PAH1*::*HIS3MX6*), and *dga1Δlro1Δ* (*DGA1*::*kanMX4*, *LRO1*::*hphNT1*). The Tet-promoter strains used for expression of essential genes under the regulatable TetO7 promoter were obtained from Open biosystems.

### Reagents

Yeast extract, peptone, dextrose, and yeast nitrogen base were purchased from BD. Nile red, choline, ethanolamine, inositol, doxycycline, cerulenin, and Ficoll 400 were from Sigma. 1, 2-dioctanoyl-sn-glycerol 3-phosphate (PA 8∶0/8∶0), 1, 2-dioleoyl-sn-glycero 3-phosphate (PA 18∶1/18∶1), oleoyl-L-α-lysophosphatidic acid, 1,2-dioctanoyl-sn-glycerol (DAG 8∶0/8∶0), phosphatidylethanolamine, phosphatidylcholine, and triolein were purchased from Avanti Polar Lipids.

To screen for yeast mutants that generate supersized LDs, cells were cultured in synthetic complete media (0.67% yeast nitrogen base, 2% dextrose, and amino acids) in 96-well plates at 30°C till stationary phase. For TetO7-regulated strains, 15 µg/ml doxycycline was added to repress specific genes. Cells were stained with 20 µg/ml Nile red for LDs and observed by fluorescence microscopy. Yeast strains found to contain SLDs were recultured in synthetic complete media with aeration in 10 ml culture tubes to confirm the phenotype. These strains were also grown in YPD media (1% yeast extract, 2% peptone, and 2% dextrose) to examine the morphology of LDs.

For phospholipid precursor treatment, synthetic complete medium was supplemented with 1 mM choline, 1 mM ethanolamine, or 75 µM inositol.

### Fluorescence microscopy

Fluorescent imaging was performed under a Leica CTR5500 microscope (Wetzlar, Germany) with an EL6000 fluorescent lamp. Images were taken with a DFC300 FX digital camera and a Leica LAS AF software. Yeast cells were viewed under a×100/1.30 oil immersion objective lens. A 450−490-nm bandpass excitation filter, a 510 dichromatic mirror, and a 515-nm longpass emission filter (Leica filter cube I3) were chosen to observe Nile red-stained LDs. For statistical presentation of the percentage of cells containing supersized LDs, 200 cells were observed and percentage was calculated. The experiments were done in triplicates and the result was shown as mean ± SD. Mammalian LDs were stained with Bodipy 493/503 (Invitrogen) and observed with a 470/40-nm bandpass excitation filter, a 500-nm dichromatic mirror, and a 525/50-nm bandpass emission filter (Leica filter cube GFP).

To observe and record LD fusion, 3 µl of mid-log phase cells (OD_600_∼1.5) or purified LDs were stained with Nile red, spotted on a slide and covered with a coverslip. Under the microscope, cells in which two or several LDs lay close together were targeted. Images were collected at 0.5 second intervals.

### Transmission electron microscopy

Cells were grown in rich medium until stationary phase, harvested, fixed with 2.5% glutaraldehyde and postfixed with 2% (w/v) osmium tetroxide. The samples were subsequently dehydrated in a series of graded ethanol and embedded in Spurr's Resin. 80 nm ultrathin sections were stained with uranyl acetate and lead citrate and examined under a JEM-1230 Joel electron microscope.

Total RNA was extracted using the RNeazy Plus kit (QIAGEN). cDNA was generated from total RNA using a SuperScript VILO cDNA Synthesis Kit (Invitrogen). PCR reaction was performed using Rotor-Gene RG-3000A (Qiagen). Threshold cycle value for each gene was acquired at the log phase and gene expression was normalized to reference genes as indicated. For Affymetrix Array processing and analysis, samples were prepared according to the Affymetrix GeneChip® Yeast Genome 2.0 Array protocol. Differences between WT and *fld1Δ* strains were compared using one-way ANOVA and adjusted for false discovery rate at 0.05 level. Array data is deposited on Gene Expression Omnibus (http://www.ncbi.nlm.nih.gov/geo/).

### Isolation of microsomes

Microsomes were isolated as described [42]. Briefly, WT and *fld1*Δ cells were cultured in SC media till log phase (OD600∼1.0) and harvested. Cells as 0.1 g (wet weight) /ml in 0.1 M Tris SO4 were sequentially incubated in pH9.4/10 mM DTT for 10 min, and in 1.2 M sorbitol/20 mM Tris Cl, pH7.5/1x SC medium/zymolase 100 T (15 mg/ml) for 30 min. Spheroplasts were lysed in HEPES lysis buffer (10 mM HEPES/KOH, pH6.8/50 mM potassium acetate/100 mM sorbitol/2 mM EDTA) with the aid of a Dounce homogenizer. After removal of cell dedris, lysates were centrifuged at 30 000 g for 10 min at 4°C. P30 000 g membrane pellets were resuspended in HEPES lysis buffer, loaded onto 1.2 M/1.5 M sucrose (prepared in HEPES lysis buffer) gradients, and centrifuged at 100 000 g for 1 h at 4°C. ER membranes were collected at the 1.2 M/1.5 M sucrose interface.

### Lipid droplets

Lipid droplets were isolated as described previously [12].

### Isolation and analyses of lipids

Lipids were extracted from zymolase-digested lyophilized yeast cells, isolated ER microsomes or lipid droplets. Briefly 900 µl ice-clod chlorofom:methanol (1∶2) was added to samples. Mixtures were vigorously vortexed for 1 min, and incubated for 2 h in vacuum container with rotary shaking at 4°C. Then 400 µl ice-cold water and 300 µl chloroform were added, vortexed and incubated on ice for 1 min. After centrifugation at 12000 rpm for 3 min at 4°C, the lower organic phase was collected. Subsequently, 50 µl 1 M HCl and 500 µl chloroform were added to the remainder, vortexed, and incubated on ice for 3 min. The lower organic phase was also collected after centrifugation at 12000 rpm for 3 min at 4°C, and combined with the first extract. The extracted lipids were blown dry with nitrogen gas, and resuspended in solvent for mass spectrometry analysis.

Lipidomic analysis, and quantitative measurement of neutral lipids via thin layer chromatography (TLC) were performed as described [12], [43]. TLC plates were developed in chloroform/methanol/water (65∶25∶4) to separate phospholipid species.

### *In vitro* fusion assay of artificial lipid droplets

To prepare lipid emulsions, lipids were mixed in chloroform/methanol (2∶1), dried under a stream of N2, resuspended in buffer (150 mM NaCl, 50 mM Tris/HCl, pH 7.5, 1 mM EDTA,), and sonicated. For emulsions, the molar ratio of TAG to total phospholipids was 2∶2.5. Contaminating vesicles were removed, and LDs were concentrated by ultracentrifugation at 100,000 g for 15 min. For light scattering, lipid concentration was 25 mM phospholipids and 20 mM TAG before centrifugation.

### Statistical analysis

All data are presented as mean ± SD. Statistical comparison between the two groups was performed using Student's t-test. Microarray data were analyzed using one-way ANOVA and adjusted for false discovery rate at 0.05 level.

## Supporting Information

Figure S1 Biosynthetic pathways of major phospholipids and TAG in *S. cerevisiae*. PA, phosphatidic acid; CDP-DAG, CDP-diacylglycerol; PI, phosphatidylinositol; PS, phosphatidylserine; PE, phosphatidylethanolamine; PMME, phosphatidylmonomethylethanolamine; PDME, phosphatidyldimethylethanolamine; PC, phosphatidylcholine; DAG, diacylglycerol; TAG, triacylglycerol. Grey-shaded enzymes are under the control of the Ino2p-Ino4p complex.
(TIF)

Figure S2 In vivo TAG mobilization of WT, *fld1Δ*, *ino4Δ*, and *cds1* in the presence of 10 µg/ml cerulenin. Cells were grown in SC medium for 24 hr, and refreshed in YPD medium supplemented with 10 µg/ml cerulenin to OD600∼1.5. Cells were collected at indicated time points, followed by fluorescence microscopy and lipid analysis.
(TIF)

Figure S3 The effect of *DPP1* and *PAH1* expression on cellular lipids. A) Total cellular PA as measured by mass-spectrometry. *, *p*<0.01, compared to vector control. B) Major phospholipids on lipid droplets measured by mass-spectrometry. Lipid droplets were isolated as described in Materials and Methods.
(TIF)

Figure S4 Relative cellular levels of PC, PE and PI in WT and mutants as determined by thin layer chromatography (TLC). Densitometric analysis was performed using the Image Gauge 4.0 software (Fujifilm Science Lab).
(TIF)

Video S1 *cho2Δ* display enhanced fusion activity of LDs *in vivo*. Cells were grown in SC medium until mid-log phase (OD_600_ = ∼1.5), stained with Nile red and examined for LD fusion activities by fluorescence microscopy. Cells in which two or several LDs lay close together were targeted. Images were taken at a 0.5 second interval.
(AVI)

Video S2 *ino2Δ* display enhanced fusion activity of LDs *in vivo*. Cells were grown in SC medium until mid-log phase (OD_600_ = ∼1.5), stained with Nile red and examined for LD fusion activities by fluorescence microscopy. Cells in which two or several LDs lay close together were targeted. Images were taken at 0.5 second interval.
(AVI)

Video S3 Fusion of LDs *in vitro*. *fld1Δ* cells were cultured in SC media until early stationary phase. LDs were isolated and resuspended in PBS. The mixture was then stained with Nile red and 3 µl was spotted onto a slide. Under the microscope, two or several LDs lying close together were targeted. Images were taken at a 0.5 s interval.
(AVI)

Video S4 Fusion of LDs *in vitro*. *ino2Δ* cells were cultured in SC media until early stationary phase. LDs were isolated and resuspended in PBS. The mixture was then stained with Nile red and 3 µl was spotted onto a slide. Under the microscope, two or several LDs lying close together were targeted. Images were taken at a 0.5 s interval.
(AVI)
