# Supplementary figures and images for: A Role for Phosphatidic Acid in the Formation of “Supersized” Lipid Droplets
Source: PLoS Genet. 2011 Jul 28;7(7):e1002201. doi: 10.1371/journal.pgen.1002201 (PMC3145623; doi:10.1371/journal.pgen.1002201)

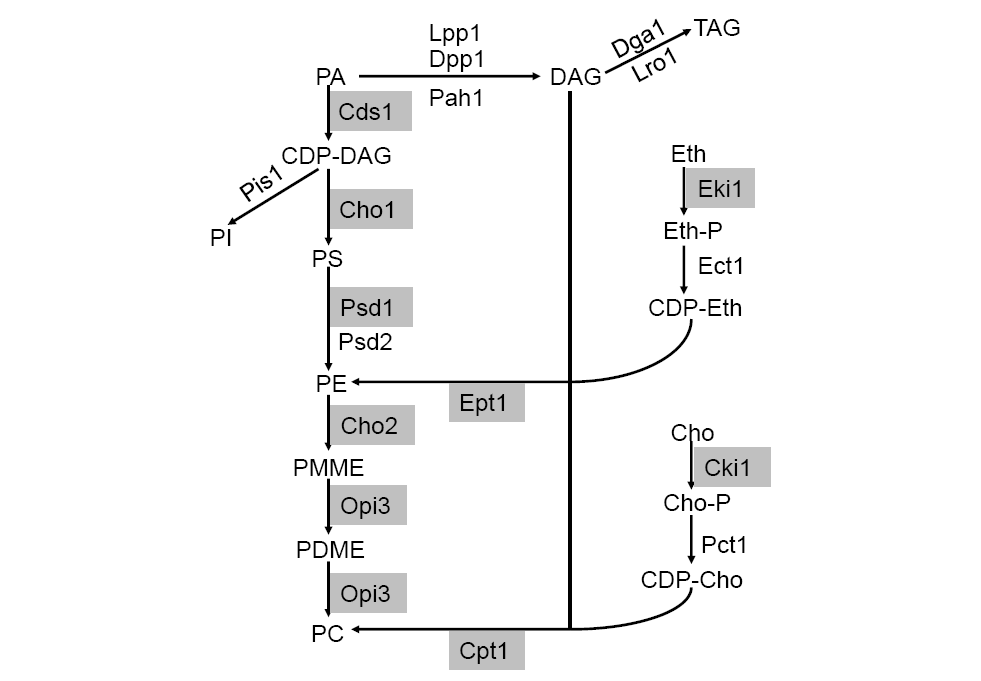

Supplement: Figure S1 — Biosynthetic pathways of major phospholipids and TAG in S. cerevisiae. PA, phosphatidic acid; CDP-DAG, CDP-diacylglycerol; PI, phosphatidylinositol; PS, phosphatidylserine; PE, phosphatidylethanolamine; PMME, phosphatidylmonomethylethanolamine; PDME, phosphatidyldimethylethanolamine; PC, phosphatidylcholine; DAG, diacylglycerol; TAG, triacylglycerol. Grey-shaded enzymes are under the control of the Ino2p-Ino4p complex. (TIF) [file pgen.1002201.s001.tif]

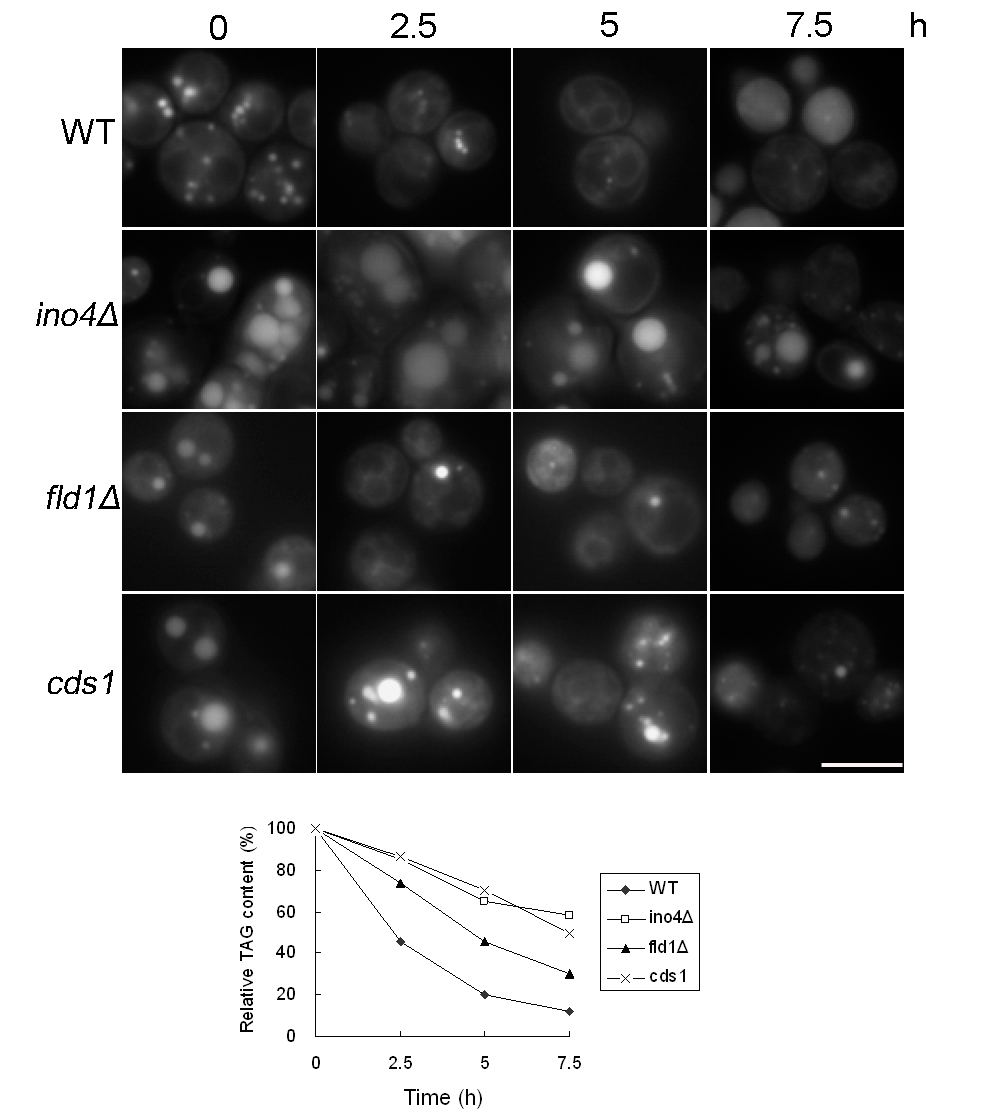

Supplement: Figure S2 — In vivo TAG mobilization of WT, fld1Δ, ino4Δ, and cds1 in the presence of 10 µg/ml cerulenin. Cells were grown in SC medium for 24 hr, and refreshed in YPD medium supplemented with 10 µg/ml cerulenin to OD600∼1.5. Cells were collected at indicated time points, followed by fluorescence microscopy and lipid analysis. (TIF) [file pgen.1002201.s002.tif]

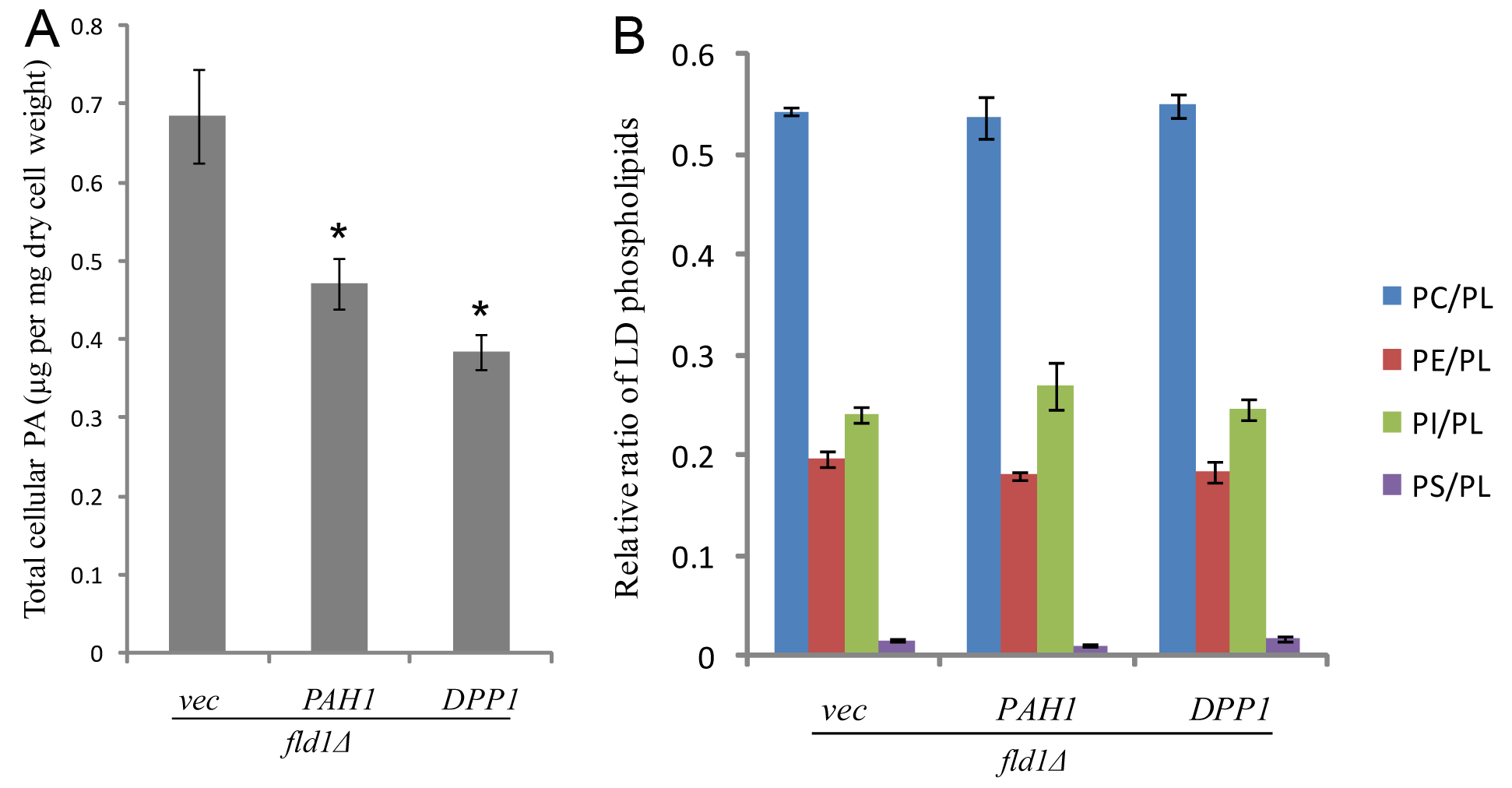

Supplement: Figure S3 — The effect of DPP1 and PAH1 expression on cellular lipids. A) Total cellular PA as measured by mass-spectrometry. *, p<0.01, compared to vector control. B) Major phospholipids on lipid droplets measured by mass-spectrometry. Lipid droplets were isolated as described in Materials and Methods. (TIF) [file pgen.1002201.s003.tif]

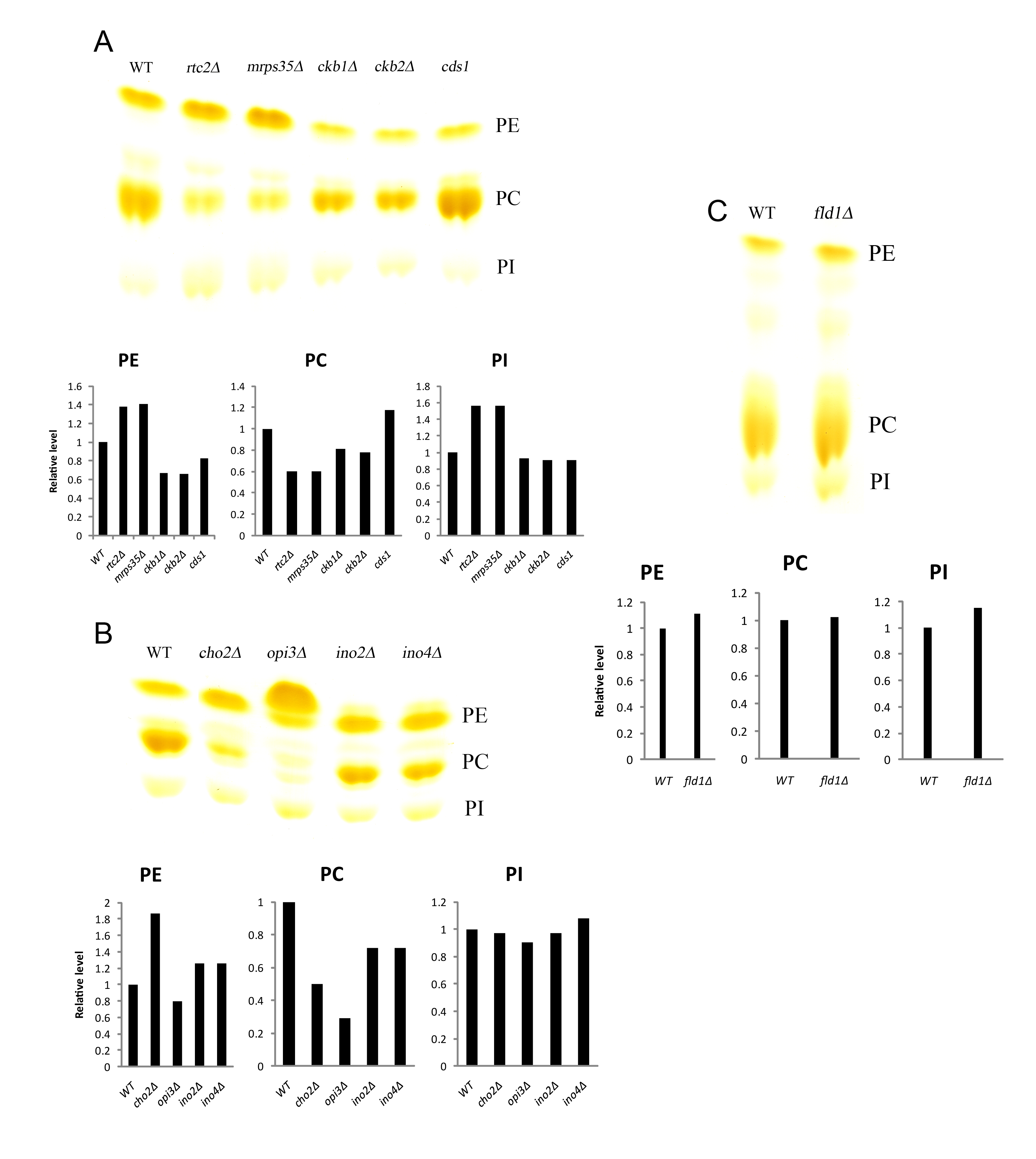

Supplement: Figure S4 — Relative cellular levels of PC, PE and PI in WT and mutants as determined by thin layer chromatography (TLC). Densitometric analysis was performed using the Image Gauge 4.0 software (Fujifilm Science Lab). (TIF) [file pgen.1002201.s004.tif]
